# Units for vigilance of emerging diseases based on wastewater treatment plants (WWTP‐UVED)

**DOI:** 10.1111/1751-7915.13635

**Published:** 2020-07-27

**Authors:** Adoración Barros‐Rodríguez, Maximino Manzanera

**Affiliations:** ^1^ Institute for Water Research and Department of Microbiology University of Granada Granada Spain

## Abstract

Pandemics deeply affect the health and economy of the world population. A precise determination of affected communities is of great importance to establish containment measures and reduce the economic impact. Here, we propose the development of Units for Vigilance of Emerging Diseases based on the screening of pathogens released to wastewater treatment plants to follow the spread of the infectious agent to determine the location of infected people.

## Abstract 2

Pandemics deeply affect the health and economy of the World population. A precise determination of affected communities is of great importance to establish containment measures and reduce the economic impact. Here, we propose the development of Units for Vigilance of Emerging Diseases (UVEDs) based on the screening of pathogens released to wastewater treatment plants to follow the spread of the infectious agent to determine the location of infected people.

The emergence of pandemics affects people's health, the economy and many other sectors of society. The emergence of viruses (COVID‐19, Zika, H1N1, H5N1, SARS, etc.), bacteria (*Vibrio cholerae, Escherichia coli, Salmonella thyphimurium*, etc.) and different types of parasites (species of *Plasmodium*, *Trypanosoma*, etc.) frequently strike our societies (Daszak *et al*., 2020). The identification of infected individuals in one part of the country can also result in overly stringent measures affecting other distant communities due to their geographical location, even when no case of infection has ever been found in the later and the infection risk is low (Gatto *et al*., 2020). These measures may include the restriction of visitors, blocking and subjection to quarantine of local inhabitants, interruption of flights and other transport of people and goods, preventive treatments such as the use of face masks, gloves or protective glasses (Feng *et al*., 2020). Curative programmes (including vaccines and drugs prescription) are also included to reduce the effect of the disease, and even the construction or adaptation of hospital facilities to respond to the demands of those affected. Frequently, health and local authorities avoid performing clinical analyses such as the use of scarce detection kits, involving qPCR, or seroprevalence surveys using tests for the detection of antibodies, that determine the extent of the contagion to prevent side effects. In many cases, the results of these analyses demonstrate the lack of rigor from authorities regarding the spread of the disease in their jurisdiction, especially by those infected people who do not suffer serious effects, but who are transmitters of the disease (London and Kimmelman, 2020). The clear determination of which communities are affected and which are free from pathogens is of great importance to establish the best containment measures without unnecessarily affecting communities free of the infectious agent. Such measures reduce damage caused by the fear of the population, and the reduction of negative economic impact, which responds with an unnecessary alarm even in unaffected areas. Therefore, specific and independent protocols are required to ensure exhaustive monitoring of the pathogen’s spread. We propose an analysis of local communities instead of, or in addition to, the scrutiny of individuals. Patients affected by a large number of infectious pathogens including SARS‐CoV‐2 release them through their faeces (Wu *et al*., 2020). These faeces are finally treated in wastewater treatment plants (WWTPs; Randazzo *et al*., 2020). The number of pathogens found in these WWTPs is proportional to the number of affected people and their degree of affection. Given that the network of households that discharge their wastewaters into each of the WWTPs is perfectly defined, the determination of pathogens found in each WWTP allows us to establish a clear map of the geographical extension of the disease and to estimate the number of individuals affected in each zone in real time.

Today, we have enough culture‐independent techniques for the extraction and massive sequencing of nucleic acids (e.g. Illumina or Ion Torrent) and through specific amplification of pathogen genes by qPCR, to monitor the unusual presence of pathogens at any population’s WWTPs. We therefore suggest the creation of Units for Vigilance of Emerging Diseases (UVEDs) based on the continuous analysis of pathogens in WWTPs in potentially affected areas and especially during epidemics and pandemics. The results potentially found by these UVEDs would allow collecting adequate information for the development of new therapeutic and preventive tools. For example, mass sequencing allows us to identify if certain microorganisms present in the wastewater carry antibiotic resistance genes or if a subpopulation may have changed the epitope recognized by a vaccine. This may allow to know the probability of the efficacy of a treatment in advance. We could avoid unnecessary expenses, unnecessary exposure to therapeutics such as antibiotics, preventing thus the occurrence of resistance and reduction in the efficacy of certain drugs. However, in order to provide a reliable service, some issues need to be addressed, such as developing an accurate nucleic acid (DNA and RNA) extraction protocol and a topological analysis of the sanitation network. For the nucleic acid extraction protocol, most of the available extraction kits are for human samples (including stools) and environmental samples such as soils, but not designed to extract RNA from wastewater samples. Wastewater are of heterogeneous nature and can be mixed with different concentrations of sanitation products such as bleach that could affect the stability of the nucleic acids and the efficiency of the extractive technique. Therefore, buffering the wastewater sample is of paramount importance prior to the nucleic acid extraction. In addition, the extraction method should equally work for bacteria and for the different viral pathogens that can be grouped into seven different categories accordingly to the type of nucleic acid they contain by the Baltimore Classification (including double‐stranded DNA, single‐stranded DNA, double‐stranded RNA, and positive‐ and negative‐single‐stranded RNA). For the topological analysis of the sanitation network, appreciation of the different discharge of wastewaters needs to be taken into consideration, since there are times when the volume of wastewater is higher and an appropriate model needs to be established to consider such variations and to be able to provide an approximate number of affected people in relation to the detected viral particles.

Most of the cost of an outbreak is not due to the pathogen itself, but due to the panic it causes. This is reflected in the fall in oil prices and the collapse of the stock markets. The WHO estimates that the expenses of this type of outbreak are approximately 40 billion euros (e.g. approximately 40 billion in 2003 for SARS and 40–50 billion for influenza A or H1N1 in 2009; Goodell, 2020).

International agencies consider that an annual investment of between 1.9 and 3.4 billion to strengthen plant, animal and human health systems would produce a global public benefit. The fact that treated wastewater is used for agriculture might potentially disseminate plant pathogens across wide agricultural areas that are worth monitoring. Current legislation in many countries requires the analysis of the presence of certain pathogens such as *Salmonella,* faecal coliforms and *E. coli*. Theses analyses are normally performed by WWTPs managing companies whose costs are normally passed on to the consumer. Therefore, we believe that the creation of such UVEDs for WWTPs under the coordination of a central service to analyse countrywide nucleic acid samples would greatly benefit our society and should be implemented by future legislation similarly to the analysis of other pathogens in the treated wastewater. This approach would represent the beginning of a shift from personalized medicine to community medicine.

## Conflict of interest

None declared.
